# Supplementary material for: Persistence of SARS-CoV-2 Viral RNA in Nasopharyngeal Swabs after Death: An Observational Study
Source: Microorganisms. 2021 Apr 10;9(4):800. doi: 10.3390/microorganisms9040800 (PMC8103507; doi:10.3390/microorganisms9040800)
Supplement: Supplementary file 1 [file microorganisms-09-00800-s001.pdf]

Table 2 reports the R^2 and p values of the Pearson's correlation perform to evaluate the possible association among the Ct values of N, R and R genes at both T1 and T3 and the serum values of PCR, neutrophils and lymphocytes.

	PCR	Neutrophils	Lymphocytes
Swab within 2 hours of death (T1)			
N ct	-0.04 (p=0.73)	0.001 (p=032)	-0.04 (p=0.99)
Rd Rp Ct	0.02 (p=0.25)	-0.04 (p=0.85)	-0.02 (p=0.46)
E ct	-0.05 (P=0.97)	0.002 (p=0.32)	0.02 (p=0.22)
Swab 24 hours after death (T3)			
N ct	-0.06 (p=0.69)	0.09 (p=0.11)	-0.06 (p=0.49)
Rd Rp Ct	0.06 (p=0.18)	0.07 (p=0.14)	-0.06 (p=0.96)
E ct	0.04 (p=0.22)	0.09(p=0.13)	-0.06 (p=0.98)
